# Left Hemiclamshell Approach for Posterior Aortic Arch Aneurysm

**DOI:** 10.1055/s-0040-1715469

**Published:** 2021-03-19

**Authors:** Alexander Moiroux-Sahraoui, Pascal Leprince, Pierre Demondion

**Affiliations:** 1 Department of Cardiac Surgery, Sorbonne Université, Pitié-Salpêtrière Hospital, Assistance Publique-Hôpitaux de Paris, Paris, France

**Keywords:** hemiclamshell, aortic arch aneurysm, ductus arteriosus

## Abstract

The anatomical situation of posterior aortic arch aneurysms is a surgical challenge. The surgical approach should not only guarantee a safe dissection of the supra-aortic trunks and the descending aorta but also allow the cannulation for extracorporeal circulation. Indeed, protecting the cerebral flow is essential. Another challenge is to preserve both the phrenic and recurrent nerves while sparing chest wall muscles. A hemiclamshell approach for posterior aortic arch aneurysm seems to be a good compromise regarding these requirements.

## Introduction

The anatomical relations of posterior aortic arch aneurysms force us to address the issue of the proper surgical approach. 1 In fact, the incision should guarantee a safe dissection of the supra-aortic trunks and the descending aorta and also allow the cannulation for extracorporeal circulation (ECC). Protecting the cerebral flow and circulation downstream to the aortic clamping is yet another challenge that this pathology dictates. Another challenge is to preserve both the left phrenic and recurrent laryngeal nerves, while sparring a maximum of the chest wall muscles. We present here a left hemiclamshell approach for posterior aortic arch aneurysms.

## Techniques and Results

We report our strategy regarding two cases of posterior aortic arch aneurysms. Both cases were discovered incidentally and involved young male patients (36 and 26 years old).

**Fig. 1:**
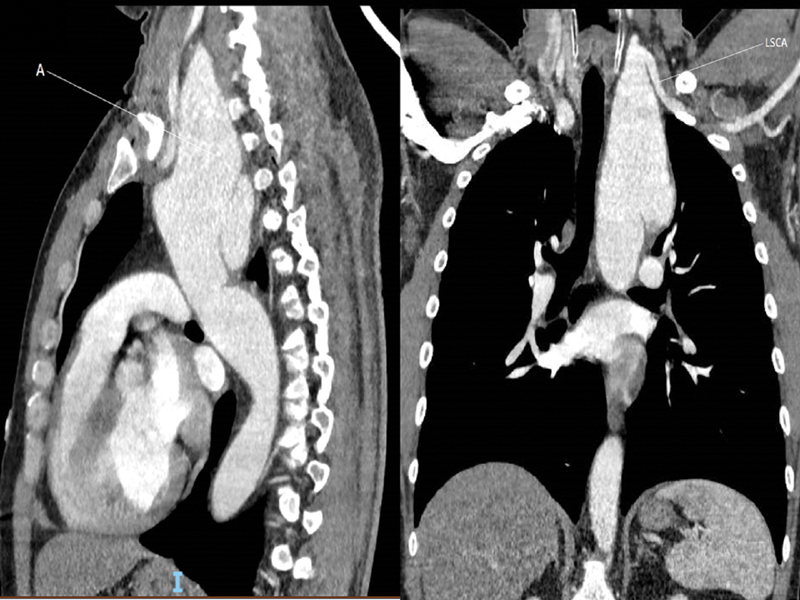
Computed tomography scan of patient 1. A,aneurysm ; LSCA,left subclavicular artery.

**Fig. 2:**
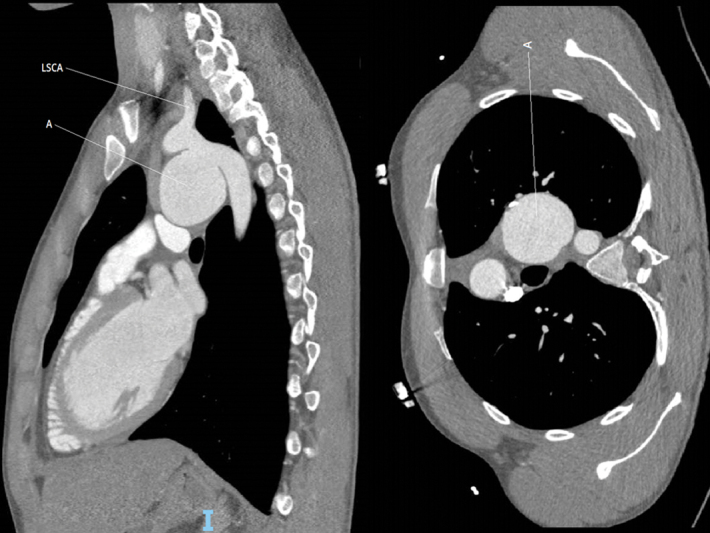
Computed tomography scan of patient 2. A, aneurysm; LSCA, left subclavicular artery.

For patient 1, the involvement of the left subclavian artery made endovascular aneurysm repair (EVAR) impossible ( Fig. 1 ). For patient 2, it was the short landing zone that contraindicated EVAR (landing zone < 1 cm; Fig. 2 ).

Hence we proceeded with therapy by direct open surgical repair of those two posterior aortic arch aneurysms using the left hemiclamshell approach.

The patients were placed in supine position with a log lifting their left hemithorax. The left lung was excluded from ventilation. The approach consisted of a partial vertical median sternotomy extended into an anterior thoracotomy in the fourth intercostal space. The left mammary pedicle was ligated and severed near the fourth intercostal space. To facilitate exposure, we cut through the posterior arch of the first rib. The posterior arch of the left first rib is transected from inside the thorax with a standard rib cutter.

**Fig. 3:**
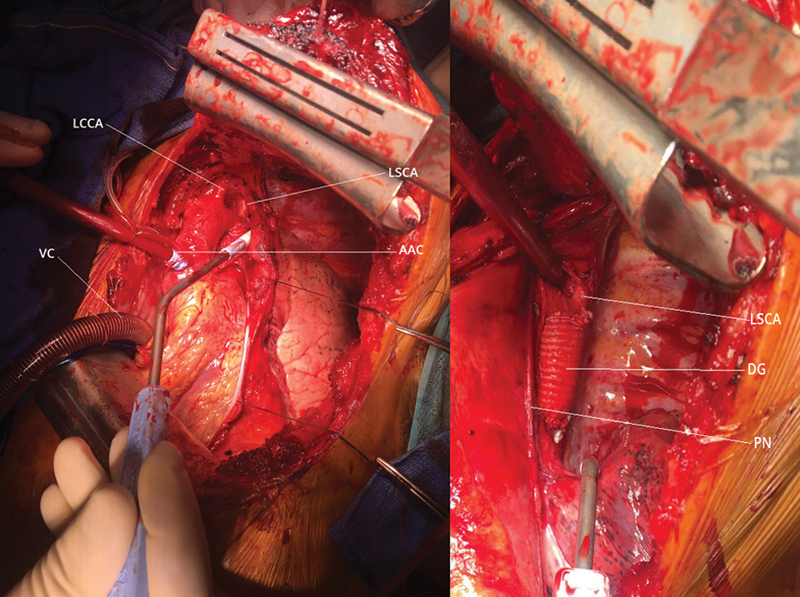
Patient 2, intraoperative view. AAC, ascending aota cannulation; DG, Dacron graft; LCCA,left common carotid artery; LSCA, left sub-clavicular artery; PN, phrenic nerve; VC,venous cannula.

The surgery followed with the dissection of the supra-aortic trunks, the descending aorta, the left phrenic nerve, and the left recurrent nerve ( Fig. 3 ). When working in close proximity with the aneurysm, extracorporeal circulation was used.

ECC consisted of an atriocaval cannulation through the right atrium and a double arterial cannulation (femoral artery and descending aorta [ Fig. 3 ]). The technique answered to the need for protecting the cerebral flow and the circulation downstream to the aortic clamping during luxating the heart. No cardioplegia was used during ECC. Proximal aortic clamping was done between the left carotid artery and the left subclavian artery. Distal clamping was done on the descending aorta, a few centimeters downstream from the aneurysm. The left subclavian artery was clamped separately.

For patient 1, the aneurysm sack was spared but two intercostal arteries were ligated, whereas patient 2 benefited from a total resection of the aneurysm. Since no intercostal artery emerged from the aneurysm, we did not have to ligate any of them.

**Fig. 4:**
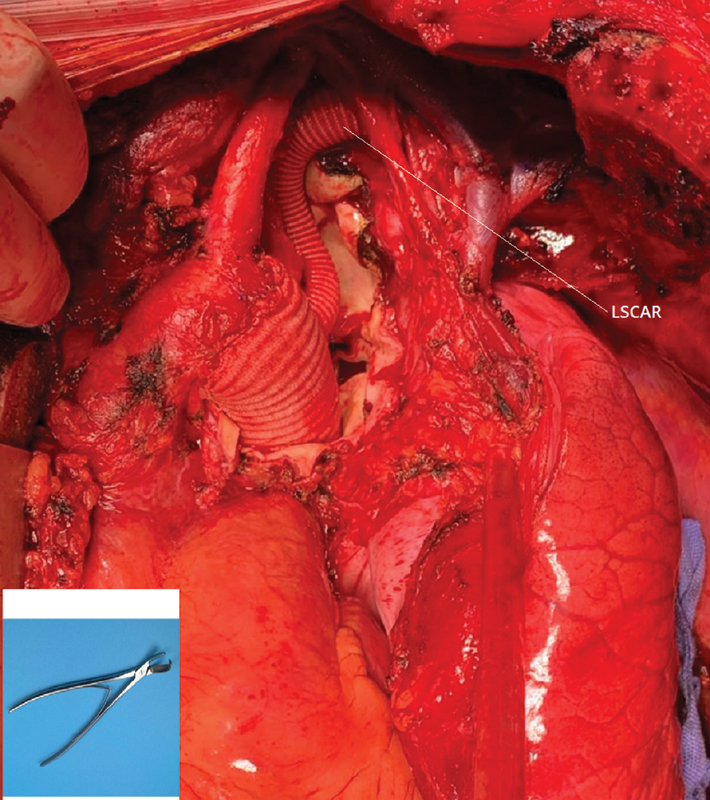
Patient 1, intra operative view and the rib cutter. LSCAR,left sub-clavicular artery reimplantation.

Dacron prostheses were used to reestablish aortic continuity (size 26 mm for patient 1 and size 20 mm for patient 2). Reimplantation of the left subclavian artery was necessary for patient 1 ( Fig. 4 ).

Cardiopulmonary bypass and aortic clamping times were 142 and 108 minutes for patient 1 and 86 and 49 minutes for patient 2, respectively.

Patient 2 exited the operating room with two analgesic catheters on both sides of the incision.

**Fig. 5:**
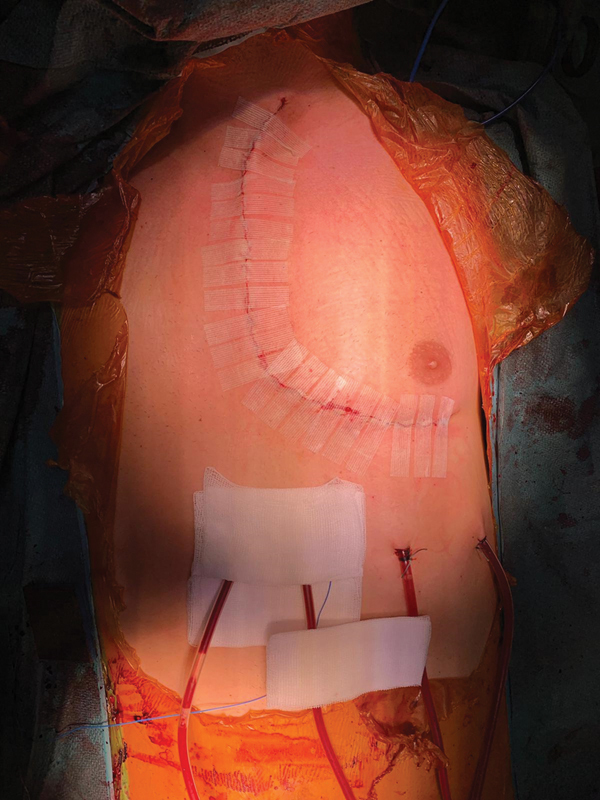
Postoperative wound.

Surgical outcomes consisted of a transient diaphragmatic paresis in patient 1. No laryngeal paralysis was observed. Postoperative computed tomography did not show any anastomotic pseudoaneurysms. Wounds were clean and no skin infection was noted ( Fig. 5 ).

## Discussion

In both the cases, the suspected etiology is ductus arteriosus aneurysm. There was no history of thoracic trauma or infection to contradict this hypothesis. 2 Unlike the case by Buechner et al, 3 EVAR was not practicable for the reasons listed above (i.e., the damage of the left subclavian artery for patient 1 and the short landing zone for patient 2).

Left hemiclamshell approach allows double arterial cannulation (femoral artery and descending aorta) for the purpose of protecting the cerebral flow and the circulation downstream the aortic clamping during luxating the heart for the dissection of the posterior mediastinum. It also offers a satisfying operative view of all the key anatomical structures to operate in good and secure conditions, while limiting the risks of nerve lesions (phrenic and recurring nerves) and muscle infringement (latissimus dorsi, serratus anterior, trapezius, and rhomboid major). Postoperative pain and rehabilitation ensue from the respect of these structures.

For patient 2, posterolateral thoracotomy could have been contemplated. However, the previous requirements (nerve and muscle sparring) and the need for perfect access to the aortic cross between the left carotid artery and the left subclavicular artery kept us away from that approach.

In conclusion, the left hemiclamshell approach for posterior aortic arch aneurysm seems to be a good compromise regarding all the requirements listed above.
